# A Contemporary Review of the Treatment Landscape and the Role of Predictive and Prognostic Biomarkers in Pancreatic Adenocarcinoma

**DOI:** 10.1155/2018/1863535

**Published:** 2018-02-12

**Authors:** Irene S. Yu, Winson Y. Cheung

**Affiliations:** ^1^Division of Medical Oncology, Department of Medicine, British Columbia Cancer Agency, University of British Columbia, Vancouver, BC, Canada; ^2^Section of Medical Oncology, Department of Oncology, Tom Baker Cancer Centre, University of Calgary, Calgary, AB, Canada

## Abstract

Pancreatic cancer continues to represent one of the leading causes of cancer-related morbidity and mortality in the developed world. Over the past decade, novel systemic therapy combination regimens have contributed to clinically meaningful and statistically significant improvements in overall survival as compared to conventional monotherapy. However, the prognosis for most patients remains guarded secondary to the advanced stages of disease at presentation. There is growing consensus that outcomes can be further optimized with the use of predictive and prognostic biomarkers whereby the former can be enriching for patients who would benefit from therapies and the latter can inform decision-making regarding the need and timing of advanced care planning. One of the challenges of current biomarkers is the lack of standardization across clinical practices such that comparability between jurisdictions can be difficult or even impossible. This inconsistency can impede widespread implementation of their use. In this review article, we provide a comprehensive overview of the contemporary treatment options for pancreatic cancer and we offer some insights into the existing landscape and future directions of biomarker development for this disease.

## 1. Background

In Canada, pancreatic cancer represents the fourth leading cause of cancer-related mortality [1]. In 2017 alone, 5,500 Canadians were diagnosed with the disease, and 4,800 of these cases resulted in death [1]. It is further projected that the number of deaths will surpass that of breast cancer in the near future [2]. Among all cancers, pancreatic cancer carries the lowest five-year overall survival rate at less than 10% [1, 3], owing largely to the advanced stage of disease at the time of diagnosis. While surgery remains the only curative modality, greater than 60% of patients with pancreatic cancer are considered unresectable at presentation [1]. Without appropriate treatments, median overall survival is estimated to be only 3 to 5 months [4]. Pancreatic tumors are classified into exocrine and endocrine subtypes. The former category constitutes approximately 90% of pancreatic malignancies and it also carries significantly worse prognosis than the latter. Advances in systemic therapies for unresectable disease in the past decade have helped to improve outcomes, but further progress is required since the prognosis for the majority of cases continues to be guarded. There is significant interest and an emerging body of evidence to support the development and use of biomarkers to help risk-stratify patients based on their outcomes and to identify subpopulations that may benefit from specific therapies. In this review article, we offer a comprehensive and contemporary review of the treatment landscape and the role of predictive and prognostic biomarkers in the setting of pancreatic cancers.

## 2. History of Systemic Therapies

In the 1950s, fluoropyrimidine-based chemotherapy was the mainstay of treatment for unresectable pancreatic adenocarcinoma (PDAC) [5]. Multiple studies have evaluated different 5-fluorouracil (5-FU) combinations, including pairing it with anthracyclines, platinums, vinca alkaloids, alkylating agents, and mitomycin. However, none of these studies showed an improvement in overall survival (OS) when compared to 5-FU alone [6, 7]. In 1997, Burris III et al. demonstrated that gemcitabine was superior to 5-FU in conferring “clinical benefit,” which was defined as better appetite, less weight loss, and lower need for pain control. Gemcitabine was also associated with a modest improvement in median OS from 4.41 months to 5.65 months (*p* = 0.0025) [8]. Because of this finding, gemcitabine was approved as the preferred first-line therapy. Subsequent trials examining various gemcitabine combinations, such as with capecitabine, platinum agents, irinotecan, and pemetrexed [9–13], failed to extend survival further.

For patients with good performance status, however, Cunningham et al. showed a trend towards improvement in OS (7.1 versus 6.2 months, *p* = 0.08) and 12-month progression-free survival (PFS) (13.9 versus 8.4%, *p* = 0.004) with the use of gemcitabine plus capecitabine [9]. A post hoc analysis by Herrmann et al. supported this observation, where high functioning patients (KPS 90–100%) had statistically improved OS (10.1 versus 7.4 months, *p* = 0.014) [14]. Of note, uptake of this combination has not been high, possibly because of worse toxicities.

Likewise, a phase III National Cancer Institute of Canada randomized controlled trial investigated the addition of erlotinib to gemcitabine and also found a statistically significant improvement in OS (6.24 versus 5.91 months, *p* = 0.023) and PFS (3.75 versus 3.55 months, *p* = 0.004) [15]. This combination was approved, but the additional survival benefit of only 0.33 months in the context of excess adverse effects and significant increase in costs to the healthcare system have limited the widespread adoption of this regimen in most countries [16]. Hence, gemcitabine monotherapy remained for a long time the primary standard of care for unresectable PDAC.

## 3. Recent Therapeutic Advances

In the past decade, several landmark trials in the treatment of advanced PDAC have been published, renewing optimism for this disease. The PRODIGE 4 (ACCORD 11) phase II/III trial randomized 342 patients with untreated metastatic PDAC to single agent gemcitabine versus FOLFIRINOX. Median OS (11.1 months versus 6.8 months, *p* < 0.001) and PFS (6.4 versus 3.3 months, *p* < 0.001) were significantly higher in the FOLFIRINOX group although there were increased rates of grade 3/4 toxicities [17]. This landmark study altered the treatment landscape since FOLFIRINOX represented the first regimen to provide a survival benefit of over 4 months compared to gemcitabine. However, the administration of multiagent chemotherapy requires a good performance status, which proved to be challenging in this patient population where the majority can be significantly frail and debilitated by the aggressive biology that is typically associated with pancreatic cancer.

One reason why PDAC has been resistant to most conventional chemotherapy choices is that there is dense desmoplastic stroma surrounding the pancreas, which is perceived to impede chemotherapy delivery [18]. Efforts to overcome this include increasing the bioavailability of chemotherapy to the tumor site. An example of this is the use of nab-paclitaxel, which represents an albumin-bound formulation of paclitaxel. Nab-paclitaxel has shown antitumor activity in different malignancies that overexpress the albumin-binding protein SPARC, including cancers of the breast, lung, and skin [19–21]. In the phase I/II trial by Von Hoff et al., nab-paclitaxel combined with gemcitabine showed a median OS of 12.2 months and a 1-year survival rate of 48% [22]. The subsequent phase III MPACT study randomized 861 patients to nab-paclitaxel plus gemcitabine versus gemcitabine alone. The addition of nab-paclitaxel improved both median OS (8.5 versus 6.7 months, *p* < 0.001) and PFS (5.5 versus 3.7 months, *p* < 0.001) [23]. Tumor response rates were also higher for the combination (23% versus 7%, *p* < 0.001). Cumulative toxicities of the doublet regimen were higher, namely, in terms of myelosuppression (38 versus 27%) and peripheral neuropathy (17 versus 1%). Despite these limitations, the combination of gemcitabine and nab-paclitaxel became another first-line option for patients, particularly for the many patients who were deemed to be ineligible or unfit to receive the more potent FOLFIRINOX.

With the recent introduction of FOLFIRINOX and gemcitabine plus nab-paclitaxel into the treatment paradigm, there is growing interest in developing second-line therapies. The NAPOLI trial compared the novel agent nanoliposomal irinotecan (MM-398) versus 5-FU/leucovorin versus nanoliposomal irinotecan plus 5-FU/leucovorin in patients who failed first-line gemcitabine. MM-398 is a formulation in which irinotecan is encapsulated into liposomal-based nanoparticles, with the goal of prolonging the active drug circulation time. The authors concluded that combination nanoliposomal irinotecan plus 5-FU/leucovorin significantly improved median OS (6.1 versus 4.2 months, *p* = 0.012) and PFS (3.1 versus 1.5 months, *p* = 0.0001) compared to 5-FU/leucovorin alone [24]. Single agent nanoliposomal irinotecan did not significantly improve outcomes. Based on these results, nanoliposomal irinotecan in combination with 5-FU/leucovorin has recently been approved for second-line treatment of patients who experience disease progression or intolerance to gemcitabine.

## 4. The Need for Biomarkers

Currently, either FOLFIRINOX or gemcitabine plus nab-paclitaxel is used in the first-line treatment setting for advanced PDAC. Most clinicians will base their treatment selection on a number of factors, including comorbidities, performance status, and patient preference. However, the decision can be frequently difficult since there are very few tools that can accurately predict response to the chosen therapy. Likewise, the prognosis of patients can vary to some degree and treatment selection can be influenced by the patients' anticipated lifespan. Thus, there is a growing need to explore predictive and prognostic biomarkers to aid in the personalized treatment of PDAC. This will become increasingly relevant as more therapeutic options are developed and introduced.

The National Cancer Institute defines a biomarker as “a biological molecule found in blood, other body fluids, or tissues that is a sign of normal or abnormal process, or of a condition or disease” [25]. Biomarkers encompass a variety of molecules, including peptides, proteins, nucleic acids, and antibodies. Multiple clinical uses of biomarkers in oncology have been described. A biomarker is defined as predictive if the treatment effect varies for biomarker-positive patients compared to biomarker-negative patients [26]. In contrast, prognostic biomarkers provide information on a likely cancer outcome independent of treatment received and can be applied even in the absence of any treatment [26]. Currently, there are no FDA-approved predictive or prognostic biomarkers for PDAC even though there is a significant clinical need to identify and validate these to define subpopulations of patients that will likely benefit from a specific therapy or patients that may require an urgent intervention to avoid negative outcomes.

## 5. Landscape of Predictive Biomarkers

### 5.1. Microsatellite Instability

Microsatellite instability (MSI) is a hypermutable phenotype with a predisposition to genetic mutations due to a deficient mismatch repair (dMMR) system. The literature has supported the use of MSI status as a predictive and prognostic biomarker in colorectal cancer (CRC). MSI-high (MSI-H) patients are shown to not derive benefit from adjuvant 5-FU [27, 28]. They also have lower rates of recurrence, delayed time to recurrence, and improved survival compared to their proficient MMR counterparts [29]. Approximately 15% of CRC tumors are dMMR; this contrasts the less than 1% of sporadic PDAC [30, 31]. More recently, MSI status has been utilized to predict response to immunotherapy agents in the treatment of metastatic CRC. Both pembrolizumab and nivolumab achieved significant objective response and disease control rates [32–34] and have been approved for use after failing conventional chemotherapy in metastatic CRC with dMMR. Ipilimumab was evaluated in a phase II trial for unresectable PDAC, but it failed to show tumor response in 27 patients [35], but patients were not explicitly tested for MMR status. Recently, the indication for pembrolizumab has been extended to all MSI-H unresectable or metastatic solid tumors after progression on conventional therapies, including PDAC. This decision was based on the study by Le et al., which enrolled 86 patients with 12 different tumor types and included some PDAC cases. Objective radiographic responses were achieved in 46 (53%) patients, and complete responses were seen in 18 (21%) patients; median PFS and OS were not reached in the latest publication [36]. There were 8 (9%) pancreatic cases; 5 (62%) patients showed an objective response rate and 6 (75%) showed disease control. Despite the small sample size, this presents an appealing option for the treatment of dMMR PDAC, underscoring that further studies are warranted. However, the small proportion of pancreatic tumors that are MSI-H in the general population will likely limit the impact of this biomarker.

### 5.2. BRCA Mutations

BRCA1/2 are tumor suppressor genes involved in the repair of double-stranded DNA breaks via homologous recombination. Defective BRCA genes lead to the use of alternative low-fidelity repair pathways and have been associated with the development of multiple malignancies including breast, ovarian, prostate, and pancreatic cancers [37–40]. The Breast Cancer Linkage Consortium estimated the overall relative risk of PDAC to be 2.26 and 3.51 higher than the general population for BRCA1 and BRCA2 mutation carriers, respectively [41, 42]. There is a growing body of evidence for BRCA's role as a predictive biomarker for response to platinum agents and poly(ADP-ribose) polymerase (PARP) inhibitors. Platinum agents cause cross-linking of DNA to induce double-stranded DNA breaks, which are ineffectively repaired in those with a BRCA mutation. PARP aids in the repair of single-stranded DNA breaks and nucleoside base damage; PARP inhibition leads to the transformation of single-stranded breaks into double-stranded breaks. Similarly, this is hypothesized to be cytotoxic in cells with defective BRCA repair mechanisms.

The addition of platinum agents to gemcitabine has previously been reported to be ineffective in extending survival compared to gemcitabine alone in multiple studies [10, 43, 44]. Sonnenblick et al. described the use of cisplatin plus gemcitabine in a PDAC patient with a BRCA2 1153 insertion T mutation. There was initial progression on gemcitabine monotherapy, but the addition of cisplatin resulted in a complete radiographic and serologic (CA19-9) response [45]. A retrospective review by Lowery et al. studied 15 patients with BRCA mutations and locally advanced or metastatic PDAC; five of six patients receiving first-line platinum chemotherapy showed partial or complete response by RECIST criteria [46]. Subsequently, Golan et al. performed a review with 71 patients with BRCA mutations and observed a superior OS for those with stage III/IV disease treated with platinum compared to nonplatinum chemotherapy (22 versus 9 months, *p* = 0.039) [47].

Similarly, Fogelman et al. illustrated a case where iniparib, a PARP-1 inhibitor, was used in combination with gemcitabine in a patient with a known BRCA mutation. The patient initially had her disease resected followed by adjuvant chemoradiation; subsequently, her disease recurrence was treated with 3 cycles of iniparib plus gemcitabine and achieved complete pathologic response [48]. A phase II trial by Kaufman et al. enrolled a spectrum of BRCA1/2-associated cancers to investigate the safety and efficacy of the PARP inhibitor olaparib; 23 PDAC cases were included. A response rate of 22% was demonstrated in heavily pretreated patients with metastatic PDAC, with an average of two prior lines of treatment [49]. Median OS and PFS were 9.8 and 4.6 months, respectively [49].

Domchek et al. presented RUCAPANC at the 2016 ASCO Annual Meeting, which included 19 BRCA-mutated patients treated with rucaparib. Objective response rate was 11% and disease control rate (defined as partial response or stable disease > 12 weeks) was 32% [50]. There was a suggestion that individuals less heavily pretreated derived more benefit as all responders only received one prior line of chemotherapy.

With the growing evidence for the role of platinum agents and PARP inhibitors in this patient subgroup, the National Comprehensive Cancer Network (NCCN) has recommended consideration of a first-line platinum-based regimen for advanced PDAC in the setting of a BRCA mutation [51]. The ongoing phase III POLO trial is investigating the effectiveness of maintenance olaparib monotherapy after platinum therapy and may provide further direction on the use of PARP inhibitors in PDAC.

### 5.3. SPARC

Secreted protein acidic and rich in cysteine (SPARC), also known as osteonectin, is an albumin-binding protein involved in mediating interactions between cells and their extracellular environment during morphogenesis, tissue remodeling, and angiogenesis [52]. SPARC is frequently upregulated in tumor tissue and it is implicated to play a role in cancer development and growth. In PDAC, SPARC is hypothesized to facilitate the effective uptake of nab-paclitaxel. In a phase I/II trial by Von Hoff et al. investigating the safety and efficacy of gemcitabine plus nab-paclitaxel, SPARC levels were also evaluated. SPARC status was available for thirty-six patients who were divided into high- and low-level SPARC groups; a higher median OS was observed in the high SPARC cohort (17.8 versus 8.1 months, *p* = 0.0431) [22]. SPARC found in pancreatic stromal tissue correlated with OS but not SPARC derived from tumor cells. Nab-paclitaxel markedly depleted tumor stroma, while significantly enhancing intratumoral gemcitabine concentrations by 2.8-fold. Overall, nab-paclitaxel was thought to target stromal SPARC to incite an antidesmoplastic response to effectively deliver gemcitabine to the tumor site.

In a post hoc analysis, Hidalgo et al. explored the relationship between SPARC and clinical outcomes using data from the phase III MPACT trial. Contrary to prior evidence, SPARC levels from the stroma, tumor epithelium, and plasma were not found to be predictive of the overall response rate to nab-paclitaxel and gemcitabine [53]. Currently, PDAC samples are not routinely sent for SPARC testing and further studies are required to better characterize the role of SPARC as a predictive biomarker for response to nab-paclitaxel and gemcitabine. Unfortunately, there is a lack of standardization in grading SPARC expressivity, which may have influenced the seemingly contradictory study findings.

### 5.4. Human ENT1

Human equilibrative nucleoside transporter 1 (hENT1) aids in the movement of nucleosides across the cell membrane for nucleotide synthesis via the salvage pathway. Both gemcitabine and 5-FU are nucleoside analogues. Thus, nucleoside transporter expression levels may influence their uptake into tumor cells. Mohelnikova-Duchonova et al. demonstrated that PDAC cell lines have decreased levels of hENT1 mRNA compared to nonmalignant pancreatic tissue [54], which may contribute to the intrinsic chemorefractory nature of PDAC. In vitro studies have shown that increased hENT1 expression can play a role in intratumoral gemcitabine and 5-FU levels, although findings have been conflicting and other intracellular processes may be at play [55, 56]. Prior studies have shown signals whereby high levels of hENT1 in PDAC tumors that were treated with adjuvant gemcitabine predicted longer survival [57, 58], but the same did not apply to 5-FU treated patients [59]. Neoptolemos et al. investigated the therapeutic predictability of hENT1 utilizing tumor samples from the adjuvant ESPAC1/3 trials; 352 patients who received either gemcitabine or 5-FU were included. Survival between the two groups was similar, but the low-hENT1 gemcitabine group had lower survival compared to the high-hENT1 cohort (17.1 versus 26.2 months, *p* = 0.002) [60]. There was no survival benefit between low- and high-hENT1 expression groups in patients who received 5-FU.

The data on the use of hENT1 as a biomarker in the metastatic setting have been less robust. Giovannetti et al. demonstrated that high-hENT1 expression was associated with significantly longer median OS (22.34 versus 12.42 months, *p* < 0.001) and disease-free survival (DFS) (20.43 versus 9.26 months, *p* < 0.05) in those who received gemcitabine, but the patient population was heterogenous with approximately 50% of patients having stage III/IV disease [61]. In a large phase II study conducted by Poplin et al., no difference in OS was observed between the low-hENT1 and the high-hENT1 groups who received gemcitabine [62]. Overall, hENT1 testing is limited to the clinical trial setting, but it may play a role in identifying patients who will derive benefit from adjuvant gemcitabine therapy; less evidence is available for the palliative setting.

### 5.5. Human CNT3

Human concentrative nucleoside transporter 3 (hCNT3) is another cell membrane transporter that assists in the uptake of gemcitabine intracellularly against the concentration gradient. There is less evidence to support the predictive value of hCNT3 expression in estimating response to adjuvant gemcitabine compared to hENT1. Maréchal et al. studied tumor blocks from 45 PDAC patients treated with adjuvant chemoradiation consisting of gemcitabine. The high-hCNT3 group had longer median OS, which was not reached during the follow-up period, versus 12.6 months in the low-hCNT3 group; three-year survival was also higher in the high-hCNT3 group (54.6% versus 26.1%, *p* = 0.028) [63]. In a combined analysis, 15 identified patients with both high hCNT3 and hENT1 levels had longer median OS compared to those with either high hCNT3 or hENT1 levels and those with low levels of both transporters (median OS not reached versus 18.7 months versus 12.2 months, *p* < 0.0001). There may be an emerging role in combining the various nucleoside transporters as a panel of biomarkers; these will require validation in future studies. 

## 6. Landscape of Prognostic Biomarkers

### 6.1. CA19-9

CA19-9 is the most widely utilized biomarker in PDAC; it is a cell surface protein derived from the Lewis blood group antigen, which is expressed in 90 to 95% of the general population. Previous studies have mainly focused on the hypothetical role of CA19-9 in cancer screening with an estimated sensitivity and specificity of 78% and 82%, respectively [64]. ASCO guidelines currently recommend against using CA19-9 as a screening biomarker [65] as increased levels can be seen in nonpancreatic malignancies in addition to other benign conditions. Kim et al. assessed more than 70,000 asymptomatic patients with serum CA19-9 levels and abdominal ultrasound imaging. Using the cutoff value of 37 U/ml, the positive predictive value was 0.9% with only 4 cases of pancreatic cancer diagnosed [66]. Like with other biomarkers, endeavors to improve the positive predictive value of CA19-9 using higher thresholds would compromise the sensitivity of the test.

Using CA19-9 as a prognostic marker has been studied in both preoperative and postoperative settings. Ferrone et al. demonstrated that preoperative CA19-9 levels are strongly associated with final pathologic staging, with higher median CA19-9 values correlating with more advanced stages of disease [67]. In a retrospective study of patients with potentially resectable disease undergoing diagnostic laparoscopy, Maithel et al. identified a cutoff value of 130 U/ml for which levels above were predictive of occult metastatic disease by imaging. Unresectable disease was identified in 26% and 11% of patients with CA19-9 levels ≥ 130 U/ml and < 130 U/ml, respectively [68]. Hartwig et al. reviewed more than 1500 patients who underwent surgery and found the degree of preoperative CA19-9 elevation correlated with tumor resectability and survival rates. Comparing the low CA19-9 (<37 U/ml) versus high CA19-9 (≥4000 U/ml) cohorts, resectability was 80% versus 38% and median OS was 28.5 versus 14.4 months, respectively [69]. This is supported by Montgomery et al. who described a median OS of 34 versus 16 months using the preoperative threshold of 1052 U/ml [70]. In the postoperative setting, greater median survival has been shown in those with low postoperative CA19-9 levels (36.8 versus 14.6 months, *p* < 0.0001), using 37 U/ml as the threshold [69]. Similarly, Ferrone et al. observed that both an absolute decrease in CA19-9 and a cutoff of < 200 U/ml were predictive of overall survival [67].

Overall, CA19-9 is clinically used for serial monitoring after potentially curative surgery and for those on systemic therapy for unresectable disease, with the goals of detecting recurrence and assessing disease response. One limitation to the above studies is the variability in CA19-9 thresholds. Efforts to standardize the cutoff values used should be one of the emphases in future studies. CA19-9 levels generally correlate with the burden of disease and thus CA19-9 levels have also shown promise in aiding clinicians in the preoperative evaluation for resectability.

### 6.2. Clinical Factors (Nodal Status, Overall Stage, and Surgical Margins)

For individuals who have undergone resection of their PDAC, studies have shown that lymph node metastasis is associated with worse prognosis, although data are conflicting [71–75]. This may be due to the bias of incomplete lymphadenectomy at the time of surgery or the nature of the histopathologic examination. Kang et al. investigated both the lymph node ratio and the absolute lymph node number as potential prognostic biomarkers in 398 patients who underwent surgery for PDAC. Median OS was significantly higher in those without lymph node metastasis compared to one lymph node involvement (25.4 versus 17.3 months, *p* = 0.001) [76]. In patients with N1 nodal status, the lymph node ratio and number of lymph nodes did not significantly affect prognosis. Tomlinson et al. determined that at least 15 lymph nodes are required to accurately stage node negative PDAC [77]. Other studies have evaluated the location of lymph node metastasis as a potential prognostic marker. In particular, para-aortic lymph nodes have received attention as they are considered “extraregional.” A meta-analysis by Paiella et al. found that para-aortic lymph node metastases were associated with increased mortality, regardless of regional nodal status (HR 1.85, *p* < 0.001) [78]. The authors suggested that intraoperative frozen section of para-aortic lymph nodes should be examined and taken into consideration prior to proceeding to pancreaticoduodenectomy. This is presently not the standard of practice as a staging laparoscopy and peritoneal cytology examination are often performed instead. Given the high perioperative morbidity and mortality of the procedure, identifying patients who may not derive significant benefit from surgery would have significant utility.

Overall disease stage remains the most important prognostic factor in PDAC. This was validated in the study of Bilimoria et al. who restaged over 121,000 patients from the National Cancer Database using the AJCC 6th edition guidelines in order to assess the predictive ability of the staging system on survival. For all patients, there was 5-year survival discrimination by stage (*p* < 0.0001). In patients who underwent surgery, the stage of disease predicted 5-year survival: 31.4% for stage IA, 27.2% for stage IB, 15.7% for stage IIA, 7.7% for stage IIB, 6.8% for stage III, and 2.8% for stage IV [79].

Studies have also evaluated whether surgical margin status is an independent prognostic factor for survival. Results are conflicting. The definition of an incomplete microscopic resection has been inconsistent, varying from presence of tumor cells at the surface of the resection margin versus within 1.0 mm of the margin. A large study by Yeo et al. showed that a negative resection margin is a strong predictor of long-term survival, but pancreatoduodenectomy specimens examined in the study included other pathologies in addition to PDAC [80]. Konstantinidis et al. demonstrated that patients undergoing R0 resections have improved median OS compared to R1 resections (23 versus 14 months, *p* < 0.001) [81]. However, the survival benefit of an R0 resection is lost when a tumor is found within 1 mm of the surgical margin. Subsequently, Chang et al. studied the volume of residual disease by stratifying margins by 0.5 mm increments and found that a margin clearance of 1.5 mm is important for long-term survival [82]. They suggested that margins less than this threshold may require further adjuvant locoregional therapies. Overall, evidence of a positive margin decreases survival but the consensus on the definition of a negative margin remains variable and requires special attention when interpreting studies.

### 6.3. DNA Methylation

DNA hypermethylation is an epigenetic mechanism that leads to the addition of a methyl (CH_3_) residue on cytosines preceding guanosines (CpGs). It is well known that hypermethylation within promoter regions of tumor suppressor genes can lead to inactivation and contribute to oncogenesis [83]. A literature review by Henriksen et al. did not identify a hypermethylated gene that can be used on its own as a diagnostic marker for pancreatic cancer due to insufficient power [84]. Based on this study, they selected a 28-gene panel and examined promoter hypermethylation from cell-free DNA as a potential diagnostic marker. The authors found a significant difference in the mean number of methylated genes between the PDAC and control groups (8.41 versus 4.74, *p* < 0.001); their diagnostic prediction model had a sensitivity of 76% and specificity of 83% [85]. Recently, the same group utilized the panel of genes to explore the relationship between DNA methylation and stage of disease. The number of hypermethylated genes was similar for stages I–III and higher for stage IV (7 versus 10). However, their prediction model differentiated stage I-II from stage III-IV disease with a sensitivity of 73% and specificity of 80% [86]. The numbers are not ideal for clinical use but this represents a promising potential for blood-based prognostic markers. More recently, there is emerging interest in investigating DNA methylation to predict survival. Yokoyama et al. examined the effect of MUC1/4 gene methylation status on overall survival. Mucins (MUC) are transmembrane proteins that are overexpressed in different cancers and have been linked to poorer prognosis. The authors found that low methylation of MUC 1 and/or MUC 4 correlated with decreased overall survival in PDAC [87]. Additional research to identify relevant hypermethylated genes in PDAC is anticipated, especially since they may also serve as potential targets for therapeutic intervention.

### 6.4. BRCA

Unlike breast cancer, the data on using BRCA mutational status as a prognostic factor in pancreatic cancer is not well established in the literature. A recent retrospective analysis by Golan et al. included 25 cases of resected BRCA-associated PDAC and found no difference in median OS and DFS between cases and controls. There was a trend towards increased DFS in BRCA-positive patients who received a platinum agent (39.1 versus 12.4 months, *p* = 0.255) [88]. Recently, a study was published investigating polymorphisms in tumor suppressor genes to explore any effects on survival. Zhu et al. identified a specific BRCA1 missense variant that is associated with a poorer prognosis (median OS 7.5 months versus 6.7 months) [89]. Despite this, there is not enough evidence at this time to support the use of BRCA as a prognosticator of outcomes, with the exception of its potential utility in predicting response to platinum and PARP inhibitor therapies.

### 6.5. SPARC

The role of SPARC in carcinogenesis is complex and variable for different tumor sites. The lack of SPARC expression in CRC is a poor prognostic factor [90] whereas high SPARC expression in breast, melanoma, and gastric cancers is associated with worse outcomes [91–93]. For PDAC, Infante et al. reported that peritumoral SPARC expression was associated with worse prognosis when compared to the lack of SPARC expression (median survival 15 versus 30 months, *p* < 0.001) [94]. The expression of SPARC in tumor cells did not significantly affect prognosis. On the contrary, Miyoshi et al. explored the relationship between pancreatic tumor SPARC mRNA levels and prognosis; they described a higher 5-year survival for the low SPARC mRNA group compared to the high SPARC mRNA group (22.48% versus 0%, *p* < 0.0001) [95]. One limitation is that the specimens that were dissected may have included stromal tissue, as microdissection was not employed. Thus, the prognostic value of tumor SPARC expression remains uncertain.

## 7. Future Directions

There are emerging studies in the literature that are beginning to evaluate a combination of biomarkers in a panel fashion in an effort to improve the clinical value of these tests. This is particularly true in PDAC screening where there is hope that patients can be diagnosed at a much earlier stage. For instance, Zhang et al. demonstrated that CA19-9 combined with CA242 was associated with a higher sensitivity (89%) without a concomitant decrease in specificity (75%) [96]. Similarly, Brand et al. studied different circulating proteins from patients with PDAC, those with benign pancreatic disease, and healthy controls. The authors identified the combination of CA19-9, intercellular adhesion molecule 1, and osteoprotegerin to have the highest sensitivity and specificity for discriminating PDAC from healthy controls (sensitivity 88%, specificity 90%) [97]. This concept has also been extended to the prognostic setting. Kim et al. studied the combination of postoperative CEA and CA19-9 and found that patients with normal postoperative CEA levels have a longer survival even in the setting of elevated CA19-9 levels (19.1 versus 9.3 months, *p* = 0.004) [98]. In the metastatic setting, Park et al. risk-stratified patients based on performance status and certain bloodwork parameters (hemoglobin, white blood cell count, and CEA). Based on these factors, survival was found to be 11.7, 6.2, and 1.3 months for the low-, intermediate-, and high-risk groups, respectively (*p* < 0.001) [99]. Further investigations into the potential role of predictive or prognostic biomarker panels will likely improve the sensitivity and specificity of prediction and prognostication.

## 8. Conclusions

Despite advances in treatments, the prognosis of patients diagnosed with pancreatic cancer continues to be poor, due largely to the advanced stages of disease at presentation and the aggressive biology that frequently results in poor performance status and intolerance to available therapies. In order to optimize the selection of the right patients for the right therapies, there are significant efforts to develop more precise strategies which can identify subgroups of patients that will benefit from chosen treatments and those that have a limited prognosis and in whom treatments would be futile. The ultimate goal is to better tailor therapy to the individual in a move towards personalized medicine. While there have been significant gains in biomarker discovery and validation, there is still a significant gap in translating these advances from the research laboratory to the clinical bedside. One major barrier to the widespread use and implementation of existing biomarkers is the lack of standardization across jurisdictions. A collaborative and consensus-driven approach to future biomarker development may facilitate more rapid and consistent adoption of biomarkers into routine clinical practice and optimize the care that is delivered to PDAC patients.

## References

[1] Canadian Cancer Society’s Advisory Committee on Cancer Statistics (2017). *Canadian Cancer Statistics*.

[2] Ferlay J., Partensky C., Bray F. (2016). More deaths from pancreatic cancer than breast cancer in the EU by 2017. *Acta Oncologica*.

[3] Fung S., Forte T., Rahal R., Niu J., Bryant H. (2013). Provincial rates and time trends in pancreatic cancer outcomes. *Current Oncology*.

[4] Wilkowski R., Wolf M., Heinemann V. (2008). Primary advanced unresectable pancreatic cancer. *Recent Results in Cancer Research*.

[5] Teague A., Lim K. H., Wang Gillam A. (2015). Advanced pancreatic adenocarcinoma: A review of current treatment strategies and developing therapies. *Therapeutic Advances in Medical Oncology*.

[6] Cullinan S. A., Moertel C. G., Fleming T. R. (1985). A Comparison of Three Chemotherapeutic Regimens in the Treatment of Advanced Pancreatic and Gastric Carcinoma: Fluorouracil vs Fluorouracil and Doxorubicin vs Fluorouracil, Doxorubicin, and Mitomycin. *Journal of the American Medical Association*.

[7] Cullinan S., Moertel C. G., Wieand H. S. (1990). A phase III trial on the therapy of advanced pancreatic carcinoma evaluations of the mallinson regimen and combined 5‐fluorouracil, doxorubicin, and cisplatin. *Cancer*.

[8] Burris H. A., Moore M. J., Andersen J. (1997). Improvements in survival and clinical benefit with gemcitabine as first- line therapy for patients with advanced pancreas cancer: a randomized trial. *Journal of Clinical Oncology*.

[9] Cunningham D., Chau I., Stocken D. D. (2009). Phase III randomized comparison of gemcitabine versus gemcitabine plus capecitabine in patients with advanced pancreatic cancer. *Journal of Clinical Oncology*.

[10] Colucci G., Labianca R., Di Costanzo F. (2010). Randomized phase III trial of gemcitabine plus cisplatin compared with single-agent gemcitabine as first-line treatment of patients with advanced pancreatic cancer: the GIP-1 study. *Journal of Clinical Oncology*.

[11] Poplin E., Feng Y., Berlin J. (2009). Phase III, randomized study of gemcitabine and oxaliplatin versus gemcitabine (fixed-dose rate infusion) compared with gemcitabine (30-minute infusion) in patients with pancreatic carcinoma E6201: a trial of the Eastern Cooperative Oncology Group. *Journal of Clinical Oncology*.

[12] Stathopoulos G. P., Syrigos K., Aravantinos G. (2006). A multicenter phase III trial comparing irinotecan-gemcitabine (IG) with gemcitabine (G) monotherapy as first-line treatment in patients with locally advanced or metastatic pancreatic cancer. *British Journal of Cancer*.

[13] Oettle H., Richards D., Ramanathan R. K. (2005). A phase III trial of pemetrexed plus gemcitabine versus gemcitabine in patients with unresectable or metastatic pancreatic cancer. *Annals of Oncology*.

[14] Herrmann R., Bodoky G., Ruhstaller T. (2007). Gemcitabine plus capecitabine compared with gemcitabine alone in advanced pancreatic cancer: a randomized, multicenter, phase III trial of the Swiss Group for Clinical Cancer Research and the Central European Cooperative Oncology Group. *Journal of Clinical Oncology*.

[15] Moore M. J., Goldstein D., Hamm J. (2007). Erlotinib plus gemcitabine compared with gemcitabine alone in patients with advanced pancreatic cancer: a phase III trial of the National Cancer Institute of Canada Clinical Trials Group. *Journal of Clinical Oncology*.

[16] Miksad R. A., Schnipper L., Goldstein M. (2007). Does a statistically significant survival benefit of erlotinib plus gemcitabine for advanced pancreatic cancer translate into clinical significance and value?. *Journal of Clinical Oncology*.

[17] Conroy T., Desseigne F., Ychou M. (2011). FOLFIRINOX versus gemcitabine for metastatic pancreatic cancer. *The New England Journal of Medicine*.

[18] Long J., Zhang Y., Yu X. (2011). Overcoming drug resistance in pancreatic cancer. *Expert Opinion on Therapeutic Targets*.

[19] Gradishar W. J., Tjulandin S., Davidson N. (2005). Phase III trial of nanoparticle albumin-bound paclitaxel compared with polyethylated castor oil-based paclitaxel in women with breast cancer. *Journal of Clinical Oncology*.

[20] Socinski M. A., Bondarenko I. N., Karaseva N. A. (2010). Survival results of a randomized, phase 3 trial of nab-paclitaxel and carboplatin compared with cremophor-based paclitaxel and carboplatin as first-line therapy in advanced non-small cell lung cancer. *Journal of Clinical Oncology*.

[21] Hersh E. M., O'Day S. J., Ribas A. (2010). A phase 2 clinical trial of nab-Paclitaxel in previously treated and chemotherapy-naive patients with metastatic melanoma. *Cancer*.

[22] Von Hoff D. D., Ramanathan R. K., Borad M. J. (2011). Gemcitabine plus *nab*-paclitaxel is an active regimen in patients with advanced pancreatic cancer: a phase I/II trial. *Journal of Clinical Oncology*.

[23] Von Hoff D. D., Ervin T., Arena F. P. (2013). Increased survival in pancreatic cancer with nab-paclitaxel plus gemcitabine. *The New England Journal of Medicine*.

[24] Wang-Gillam A., Li C.-P., Bodoky G. (2016). Nanoliposomal irinotecan with fluorouracil and folinic acid in metastatic pancreatic cancer after previous gemcitabine-based therapy (NAPOLI-1): A global, randomised, open-label, phase 3 trial. *The Lancet*.

[25] Henry N. L., Hayes D. F. (2012). Cancer biomarkers. *Molecular Oncology*.

[26] Ballman K. V. (2015). Biomarker: Predictive or prognostic?. *Journal of Clinical Oncology*.

[27] Popat S., Hubner R., Houlston R. S. (2005). Systematic review of microsatellite instability and colorectal cancer prognosis. *Journal of Clinical Oncology*.

[28] Sargent D. J., Marsoni S., Monges G. (2010). Defective mismatch repair as a predictive marker for lack of efficacy of fluorouracil-based adjuvant therapy in colon cancer. *Journal of Clinical Oncology*.

[29] Sinicrope F. A., Foster N. R., Thibodeau S. N. (2011). DNA mismatch repair status and colon cancer recurrence and survival in clinical trials of 5-fluorouracil-based adjuvant therapy. *Journal of the National Cancer Institute*.

[30] Laghi L., Beghelli S., Spinelli A. (2012). Irrelevance of Microsatellite Instability in the Epidemiology of Sporadic Pancreatic Ductal Adenocarcinoma. *PLoS ONE*.

[31] Humphris J. L., Patch A. M., Nones K. (2017). Hypermutation in pancreatic cancer. *Gastroenterology*.

[32] Le D. T. (2016). Programmed death-1 blockade in mismatch repair deficient colorectal cancer. *Journal of Clinical Oncology*.

[33] Le D. T., Uram J. N., Wang H. (2015). PD-1 blockade in tumors with mismatch-repair deficiency. *The New England Journal of Medicine*.

[34] Overman M. J., McDermott R., Leach J. L. (2017). Nivolumab in patients with metastatic DNA mismatch repair-deficient or microsatellite instability-high colorectal cancer (CheckMate 142): An open-label, multicentre, phase 2 study. *The Lancet Oncology*.

[35] Royal R. E., Levy C., Turner K. (2010). Phase 2 trial of single agent ipilimumab (Anti-CTLA-4) for locally advanced or metastatic pancreatic adenocarcinoma. *Journal of Immunotherapy*.

[36] Le D. T., Durham J. N., Smith K. N. (2017). Mismatch repair deficiency predicts response of solid tumors to PD-1 blockade. *Science*.

[37] Miki Y., Swensen J., Shattuck-Eidens D. (1994). A strong candidate for the breast and ovarian cancer susceptibility gene *BRCA1*. *Science*.

[38] Ford D., Easton D. F., Bishop D. T., Narod S. A., Goldgar D. E. (1994). Risks of cancer in BRCA1-mutation carriers. *The Lancet*.

[39] Wooster R., Bignell G., Lancaster J. (1995). Identification of the breast cancer susceptibility gene *BRCA2*. *Nature*.

[40] Greer J. B., Whitcomb D. C. (2007). Role of BRCA1 and BRCA2 mutations in pancreatic cancer. *Gut*.

[41] Thompson D., Easton D. F. (2002). Cancer incidence in BRCA1 mutation carriers. *Journal of the National Cancer Institute*.

[42] The Breast Cancer Linkage Consortium (1999). Cancer risks in BRCA2 mutation carriers. *Journal of the National Cancer Institute*.

[43] Heinemann V., Quietzsch D., Gieseler F. (2006). Randomized phase III trial of gemcitabine plus cisplatin compared with gemcitabine alone in advanced pancreatic cancer. *Journal of Clinical Oncology*.

[44] Colucci G., Giuliani F., Gebbia V. (2002). Gemcitabine alone or with cisplatin for the treatment of patients with locally advanced and/or metastatic pancreatic carcinoma: A prospective, randomized phase III study of the Gruppo Oncologico dell'Italia Meridionale. *Cancer*.

[45] Sonnenblick A., Kadouri L., Appelbaum L. (2011). Complete remission, in BRCA2 mutation carrier with metastatic pancreatic adenocarcinoma, treated with cisplatin based therapy. *Cancer Biology & Therapy*.

[46] Lowery M. A., Kelsen D. P., Stadler Z. K. (2011). An emerging entity: pancreatic adenocarcinoma associated with a known brca mutation: clinical descriptors, treatment implications, and future directions. *The Oncologist*.

[47] Golan T., Kanji Z. S., Epelbaum R. (2014). Overall survival and clinical characteristics of pancreatic cancer in BRCA mutation carriers. *British Journal of Cancer*.

[48] Fogelman D. R., Wolff R. A., Kopetz S. (2011). Evidence for the efficacy of iniparib, a PARP-1 inhibitor, in BRCA2-associated pancreatic cancer. *Anticancer Reseach*.

[49] Kaufman B., Shapira-Frommer R., Schmutzler R. K. (2015). Olaparib monotherapy in patients with advanced cancer and a germline BRCA1/2 mutation. *Journal of Clinical Oncology*.

[50] Domchek S. M., Hendifar A. E., McWilliams R. R. (2016). RUCAPANC: An open-label, phase 2 trial of the PARP inhibitor rucaparib in patients (pts) with pancreatic cancer (PC) and a known deleterious germline or somatic BRCA mutation. *Journal of Clinical Oncology*.

[51] Tempero M. A., Malafa M. P., Al-Hawary M. (2017). Pancreatic adenocarcinoma, version 2.2017: Clinical practice guidelines in Oncology. *JNCCN — Journal of the National Comprehensive Cancer Network*.

[52] Lindner J. L., Loibl S., Denkert C. (2015). Expression of secreted protein acidic and rich in cysteine (SPARC) in breast cancer and response to neoadjuvant chemotherapy. *Annals of Oncology*.

[53] Hidalgo M., Plaza C., Musteanu M. (2015). SPARC expression did not predict efficacy of nab-paclitaxel plus gemcitabine or gemcitabine alone for metastatic pancreatic cancer in an exploratory analysis of the phase III MPACT trial. *Clinical Cancer Research*.

[54] Mohelnikova-Duchonova B., Brynychova V., Hlavac V. (2013). The association between the expression of solute carrier transporters and the prognosis of pancreatic cancer. *Cancer Chemotherapy and Pharmacology*.

[55] Mori R., Ishikawa T., Ichikawa Y. (2007). Human equilibrative nucleoside transporter 1 is associated with the chemosensitivity of gemcitabine in human pancreatic adenocarcinoma and biliary tract carcinoma cells. *Oncology Reports*.

[56] Tsujie M., Nakamori S., Nakahira S. (2007). Human equilibrative nucleoside transporter 1, as a predictor of 5-fluorouracil resistance in human pancreatic cancer. *Anticancer Reseach*.

[57] Maréchal R., Bachet J.-B., MacKey J. R. (2012). Levels of gemcitabine transport and metabolism proteins predict survival times of patients treated with gemcitabine for pancreatic adenocarcinoma. *Gastroenterology*.

[58] Nakagawa N., Murakami Y., Uemura K. (2013). Combined analysis of intratumoral human equilibrative nucleoside transporter 1 (hENT1) and ribonucleotide reductase regulatory subunit M1 (RRM1) expression is a powerful predictor of survival in patients with pancreatic carcinoma treated with adjuvant gemcitabine-based chemotherapy after operative resection. *Surgery*.

[59] Farrell J. J., Elsaleh H., Garcia M. (2009). Human Equilibrative Nucleoside Transporter 1 Levels Predict Response to Gemcitabine in Patients With Pancreatic Cancer. *Gastroenterology*.

[60] Neoptolemos J. P., Greenhalf W., Ghaneh P. (2013). HENT1 tumor levels to predict survival of pancreatic ductal adenocarcinoma patients who received adjuvant gemcitabine and adjuvant 5FU on the ESPAC trials. *Journal of Clinical Oncology*.

[61] Giovannetti E., Del Tacca M., Mey V. (2006). Transcription analysis of human equilibrative nucleoside transporter-1 predicts survival in pancreas cancer patients treated with gemcitabine. *Cancer Research*.

[62] Poplin E., Wasan H., Rolfe L. (2013). Randomized, multicenter, phase ii study of co-101 versus gemcitabine in patients with metastatic pancreatic ductal adenocarcinoma: Including a prospective evaluation of the role of hENT1 in gemcitabine or CO-101 sensitivity. *Journal of Clinical Oncology*.

[63] Maréchal R., Mackey J. R., Lai R. (2009). Human equilibrative nucleoside transporter 1 and human concentrative nucleoside transporter 3 predict survival after adjuvant gemcitabine therapyin resected pancreatic adenocarcinoma. *Clinical Cancer Research*.

[64] Poruk K. E., Gay D. Z., Brown K. (2013). The clinical utility of CA 19-9 in pancreatic Adenocarcinoma: Diagnostic and prognostic updates. *Current Molecular Medicine*.

[65] Locker G. Y., Hamilton S., Harris J. (2006). ASCO 2006 update of recommendations for the use of tumor markers in gastrointestinal cancer. *Journal of Clinical Oncology*.

[66] Kim J.-E., Lee K. T., Lee J. K., Paik S. W., Rhee J. C., Choi K. W. (2004). Clinical usefulness of carbohydrate antigen 19-9 as a screening test for pancreatic cancer in an asymptomatic population. *Journal of Gastroenterology and Hepatology*.

[67] Ferrone C. R., Finkelstein D. M., Thayer S. P., Muzikansky A., Fernandez-Del Castillo C., Warshaw A. L. (2006). Perioperative CA19-9 levels can predict stage and survival in patients with resectable pancreatic adenocarcinoma. *Journal of Clinical Oncology*.

[68] Maithel S. K., Maloney S., Winston C. (2008). Preoperative CA 19-9 and the yield of staging laparoscopy in patients with radiographically resectable pancreatic adenocarcinoma. *Annals of Surgical Oncology*.

[69] Hartwig W., Strobel O., Hinz U. (2013). CA19-9 in potentially resectable pancreatic cancer: Perspective to adjust surgical and perioperative therapy. *Annals of Surgical Oncology*.

[70] Montgomery R. C., Hoffman J. P., Riley L. B., Rogatko A., Ridge J. A., Eisenberg B. L. (1997). Prediction of recurrence and survival by post-resection CA 19-9 values in patients with adenocarcinoma of the pancreas. *Annals of Surgical Oncology*.

[71] Brennan M. F., Kattan M. W., Klimstra D., Conlon K. (2004). Prognostic nomogram for patients undergoing resection for adenocarcinoma of the pancreas. *Annals of Surgery*.

[72] Winter J. M., Cameron J. L., Campbell K. A. (2006). 1423 Pancreaticoduodenectomies for pancreatic cancer: a single-institution experience. *Journal of Gastrointestinal Surgery*.

[73] Lim J. E., Chien M. W., Earle C. C. (2003). Prognostic factors following curative resection for pancreatic adenocarcinoma: a population-based, linked database analysis of 396 patients. *Annals of Surgery*.

[74] Sohn T. A., Yeo C. J., Cameron J. L. (2000). Resected adenocarcinoma of the pancreas—616 patients: results, outcomes, and prognostic indicators. *Journal of Gastrointestinal Surgery*.

[75] Sperti C., Gruppo M., Valmasoni M. (2017). Para-aortic node involvement is not an independent predictor of survival after resection for pancreatic cancer. *World Journal of Gastroenterology*.

[76] Kang M. J., Jang J.-Y., Chang Y. R., Kwon W., Jung W., Kim S.-W. (2014). Revisiting the concept of lymph node metastases of pancreatic head cancer: Number of metastatic lymph nodes and lymph node ratio according to N stage. *Annals of Surgical Oncology*.

[77] Tomlinson J. S., Jain S., Bentrem D. J. (2007). Accuracy of staging node-negative pancreas cancer a potential quality measure. *JAMA Surgery*.

[78] Paiella S., Sandini M., Gianotti L., Butturini G., Salvia R., Bassi C. (2016). The prognostic impact of para-aortic lymph node metastasis in pancreatic cancer: A systematic review and meta-analysis. *European Journal of Surgical Oncology*.

[79] Bilimoria K. Y., Bentrem D. J., Ko C. Y. (2007). Validation of the 6th edition AJCC pancreatic cancer staging system: Report from the National Cancer Database. *Cancer*.

[80] Yeo C. J., Cameron J. L., Sohn T. A. (1997). Six hundred fifty consecutive pancreaticoduodenectomies in the 1990s: pathology, complications, and outcomes. *Annals of Surgery*.

[81] Konstantinidis I. T., Warshaw A. L., Allen J. N. (2013). Pancreatic ductal adenocarcinoma: is there a survival difference for R1 resections versus locally advanced unresectable tumors? What is a “true” R0 resection?. *Annals of Surgery*.

[82] Chang D. K., Johns A. L., Merrett N. D. (2009). Margin clearence and outcome in resected pancreatic cancer. *Journal of Clinical Oncology*.

[83] Kulis M., Esteller M. (2010). DNA methylation and cancer. *Advances in Genetics*.

[84] Henriksen S. D., Madsen P. H., Larsen A. C. (2017). Promoter hypermethylation in plasma-derived cell-free DNA as a prognostic marker for pancreatic adenocarcinoma staging. *International Journal of Cancer*.

[85] Henriksen S. D., Madsen P. H., Krarup H., Thorlacius-Ussing O. (2015). DNA hypermethylation as a blood-based marker for pancreatic cancer: A literature review. *Pancreas*.

[86] Henriksen S. D., Madsen P. H., Larsen A. C. (2016). Cell-free DNA promoter hypermethylation in plasma as a diagnostic marker for pancreatic adenocarcinoma. *Clinical Epigenetics*.

[87] Yokoyama S., Higashi M., Kitamoto S. (2016). Aberrant methylation of MUC1 and MUC4 promoters are potential prognostic biomarkers for pancreatic ductal adenocarcinomas. *Oncotarget*.

[88] Golan T., Sella T., O'Reilly E. M. (2017). Overall survival and clinical characteristics of BRCA mutation carriers with stage I/II pancreatic cancer. *British Journal of Cancer*.

[89] Zhu Y., Zhai K., Ke J. (2017). BRCA1 missense polymorphisms are associated with poor prognosis of pancreatic cancer patients in a Chinese population. *Oncotarget *.

[90] Yang E., Hyun J. K., Kwi H. K., Rhee H., Nam K. K., Kim H. (2007). Frequent inactivation of SPARC by promoter hypermethylation in colon cancers. *International Journal of Cancer*.

[91] Jones C., Mackay A., Grigoriadis A. (2004). Expression profiling of purified normal human luminal and myoepithelial breast cells: identification of novel prognostic markers for breast cancer. *Cancer Research*.

[92] Wang C.-S., Lin K.-H., Chen S.-L., Chan Y.-F., Hsueh S. (2004). Overexpression of SPARC gene in human gastric carcinoma and its clinic-pathologic significance. *British Journal of Cancer*.

[93] Massi D., Franchi A., Borgognoni L., Reali U. M., Santucci M. (1999). Osteonectin expression correlates with clinical outcome in thin cutaneous malignant melanomas. *Human Pathology*.

[94] Infante J. R., Matsubayashi H., Sato N. (2007). Peritumoral fibroblast SPARC expression and patient outcome with resectable pancreatic adenocarcinoma. *Journal of Clinical Oncology*.

[95] Miyoshi K., Sato N., Ohuchida K., Mizumoto K., Tanaka M. (2010). SPARC mRNA expression as a prognostic marker for pancreatic adenocarcinoma patients. *Anticancer Reseach*.

[96] Zhang Y., Yang J., Li H., Wu Y., Zhang H., Chen W. (2015). Tumor markers CA19-9, CA242 and CEA in the diagnosis of pancreatic cancer: A meta-analysis. *International Journal of Clinical and Experimental Medicine*.

[97] Brand R. E., Nolen B. M., Zeh H. J. (2011). Serum biomarker panels for the detection of pancreatic cancer. *Clinical Cancer Research*.

[98] Kim J., Lee Y. S., Hwang I. K. (2015). Postoperative carcinoembryonic antigen as a complementary tumor marker of carbohydrate antigen 19-9 in pancreatic ductal adenocarcinoma. *Journal of Korean Medical Science*.

[99] Park H. S., Lee H. S., Park J. S. (2016). Prognostic scoring index for patients with metastatic pancreatic adenocarcinoma. *Cancer Research and Treatment*.

